# Parental attitudes towards male human papillomavirus vaccination: a pan-European cross-sectional survey

**DOI:** 10.1186/s12889-015-1863-6

**Published:** 2015-07-08

**Authors:** Gitte Lee Mortensen, Marjorie Adam, Laïla Idtaleb

**Affiliations:** AnthroConsult, Fynsgade 24, 8000 Aarhus C, Denmark; Sanofi Pasteur MSD, 8, rue Jonas Salk, 69367 Lyon, Cedex 07 France; Ipsos Healthcare, 35 rue du Val de Marne, 75 628 Paris, Cedex 13 France

**Keywords:** Human papillomavirus, Male, HPV vaccination, Parental acceptability, Attitudes, Decision-making, Preventive health behaviour

## Abstract

**Background:**

Human papillomavirus (HPV) is a common sexually transmitted virus that can lead to severe diseases in both women and men. Today, HPV vaccination is offered to females only across Europe. We aimed to examine parental attitudes to HPV vaccination of their sons given brief information about HPV in both genders.

**Methods:**

A literature study on acceptability of male HPV vaccination was carried out to inform the construction of a study questionnaire. Following up on a Danish study from 2012, this questionnaire was applied in 1837 computer assisted interviews with parents of sons in the UK, Germany, France and Italy. In each country, the parents were representative in terms of geographical dispersion, city size and age of sons in the household. The applied questionnaires took the varying vaccination policies and delivery systems into account. The data were analysed pooled and for each country using significant statistical tests (chi-2) with a 95 % confidence interval.

**Results:**

Approximately ¾ of parents in the UK, Germany and Italy were in favour of HPV vaccination of their sons. In France, this applied to 49 % of respondents. Favourable parents wanted to protect their sons from disease and found gender equality important. Parents in doubt about male HPV vaccination needed more information about HPV diseases in men and male HPV vaccination; Rejecting parents were generally sceptical of vaccines and feared vaccination side-effects. Parents in countries with active vaccination policies (UK and Italy) tended to trust the importance of national vaccination programmes. Parents in countries with passive vaccination strategies (Germany and France) had greater need for information from health care professionals (HCP) and public health authorities.

**Conclusion:**

Given brief information about HPV in both genders, parental acceptance of HPV vaccination of sons is as high as acceptance levels for girls. All parents should be informed about HPV to make informed decisions about HPV vaccination for their children. There is a need for joint efforts from public health authorities and HCPs to provide parents with such information.

**Electronic supplementary material:**

The online version of this article (doi:10.1186/s12889-015-1863-6) contains supplementary material, which is available to authorized users.

## Background

Female vaccination against human papillomavirus (HPV) infection is included in vaccination calendars across the EU with the primary aim to prevent cervical cancer (CC). Two HPV vaccines exist, of which quadrivalent HPV vaccination (Gardasil) protects against oncogenic HPV 16 and 18 (causing around 70 % of CC) as well as types 6 and 11 causing most cases of genital warts (GW) [1]. The bivalent vaccine (Cervarix) protects against HPV types 16 and 18. Oncogenic HPV, particularly types 16 and 18, also cause other cancers in addition to CC. HPV is involved in around 50 % of vulva, vaginal and penile cancers, 90 % of anal cancers, and 60–70 % of oro-pharyngeal cancers [2]. In Europe, approximately 340.000 cases per year of HPV vaccine preventable diseases affect men and women, respectively [3]. The number of non-cervical cancers is almost similar to CC [4].

A large clinical study has shown that quadrivalent HPV vaccine is safe and effectively reduces ano-genital HPV infection and related diseases in 16–26 year-old males [5, 6]. This vaccine has a gender neutral indication and is already recommended for use in males in the USA, Canada and Australia. While the European Centre for Disease Prevention and Control (ECDC) finds the clinical evidence on the efficacy and safety of male HPV vaccination promising, it calls for further research including studies of HPV vaccine acceptability among parents and health care providers (HCP) [7]. In the EU, Austria recommends universal HPV vaccination but has yet to implement it in a vaccination programme. A permissive recommendation for male HPV vaccination is issued in the German region of Saxony and, more recently, in Ireland [8–10].

Previous studies of attitudes towards male HPV vaccination were mainly from USA and, until recently, most have focused on the prevention of CC [11]. Overall, it has been shown that parental attitudes are important to the uptake of HPV vaccination, and that most parents view vaccination of both sexes favourably, with positive views on other vaccines, recommendations from HCP and knowledge about HPV and related disease acting as positive factors. Acceptance is related to perceptions of disease risks (susceptibility and severity) and benefits and risks of vaccination [12–15]. Males are particularly accepting of a vaccine with direct benefits for themselves. The greatest barrier towards male HPV vaccination among males, parents and HCPs is the perceived absence of direct benefits for males [11, 16, 17]. Given that HPV vaccine was mainly marketed as a CC vaccine for girls, with little reference to the sexual transmission of the virus, awareness about HPV related diseases in men and the perceived relevance of vaccinating males is low [18–25].

Following up on a study of parental attitudes towards male HPV vaccination in Denmark [17], the present study examined parental views on HPV vaccination of sons in France, Germany, Italy and the UK. The aim was to gain knowledge about drivers and barriers to European parents’ acceptance of male HPV vaccination including socio-economic factors related to acceptability. We aimed to investigate attitudes in parents of sons at an age in which girls in their country are recommended HPV vaccination in national vaccination calendars (NVC) or national immunization programmes (NIP). As the information given in connection with a survey is decisive to acceptance rates, we presented parents with brief oral information about HPV transmission and related diseases in males and females before asking them about their attitudes towards HPV vaccination of their sons. To our knowledge, this is the first study using this approach to examine parental views on male HPV vaccination across several European countries.

## Methods

A literature study was carried out examining HPV vaccine acceptability. The results guided the construction of a questionnaire to be used in interviews with parents of sons in the UK, France, Germany and Italy. The questionnaire was formulated with an aim to capture relevant issues in all four European countries with their varying health care systems and HPV vaccination delivery structures.

In the UK, HPV vaccination of 12–17 year-old girls is included in the free NIP in which vaccines are mainly delivered through a school-based programme. In Italy, 12 year-old girls are included in the free NIP, though some regions also vaccinate additional cohorts; some at a reduced price for targeted groups. In Germany, HPV vaccination of girls aged 12–17 is recommended, implying a mandatory reimbursement from the sick funds of which some also reimburse HPV vaccination of additional cohorts. In France, the recommended age for female vaccination was lowered from 14 to 11 years (with catch-up for <20 year-olds) at the beginning of 2013, with reimbursement in place by May 2013. In France, 65 % of the vaccine cost is publicly reimbursed while private health insurers cover the remaining 35 %. While the UK and Italy have organised NIPs, HPV vaccination in Germany and France is in NVCs implying opportunistic vaccination [26].

Respondents were parents of 12–17 year-old sons in the UK and Germany, parents of 12 year-old sons in Italy, and parents of 11–14 year-old sons in France. 450 telephone interviews were carried out with parents in the UK and 454 interviews in France, using computer assisted telephone interviews (CATI); 482 face-to-face interviews using the same questionnaire and computer assisted personal interviews (CAPI) were carried out in Germany, and 451 in Italy, where the number of boys in the relevant age brackets is low. The sample sizes in each country were based on the incidence rate of parents of sons in the relevant age brackets which is between 4–5 %. The purpose of this was twofold: firstly, it enabled comparison with the former study conducted among 450 Danish parents of 12–15 year-old sons [17]; secondly, a minimum of 450 respondents per country is statistically valid to highlight significant differences between countries. With a sample size of 450 respondents, the confidence interval is approximately 4.2 %. Quota sampling was applied to target this specific group of respondents who were dispersed representatively corresponding to regional and urban/rural places of residence according to the National Institute of Statistics and Economic Studies (INSEE) in France, the Office for National Statistics (ONS) in the UK, the Statistisches Jahrbuch in Germany, and the Italian National Statistics Institute (ISTAT), respectively. As such, the samples were representative of parents of sons in the relevant age groups of each country. In France, the sample was randomly extracted from purchased Yellow Pages files. In the UK, sample recruitment used 50 % omnibus and 50 % White Pages. In Germany and Italy, respondents were recruited by interviewers going door-to-door, and interviews conducted in respondents’ homes. All interviews continued until the quotas on sons’ age and geographical dispersion were reached. Interviews were carried out by Ipsos Healthcare from May 15th – June 4th 2013. No personal information was collected about the participants except from what they chose to volunteer during the interviews. The participants were assured of their full anonymity and all gave oral consent prior to commencing the interviews. As such, and due to the market research approach that was applied, the study did not require ethics committee approval in any of the countries [27–32].

**Fig. 1:**
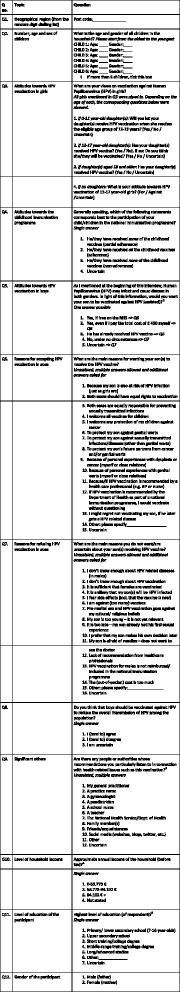
Questionnaire applied in the United Kingdom

Interviews were carried out with parents ‘having an equal or primary responsibility for health-related decisions regarding the children in the household’. Brief oral information was given to respondents who were then asked about the number and gender of children in the household, acceptance of female HPV vaccination, compliance with the NIP/NVC for children, attitudes towards male HPV vaccination to reduce the overall transmission of HPV in society, attitudes towards HPV vaccination of own son(s) and sources of recommendation mostly listened to regarding vaccination. Income level, educational level and postal code was also registered (Fig. 1. Questionnaire applied in the United Kingdom)

.

The data was analysed systematically for each individual country and pooled for the four countries. Significant statistical differences were performed with a 95 % confidence interval. Systematic analysis breakdown was performed on gender, income level, educational level, region, place of living, presence of girls in the household, HPV acceptance in girls, attitudes towards HPV vaccination to reduce the overall transmission, and sources of recommendation most listened to. Following Q5 asking about parents’ attitudes towards HPV vaccination of their sons (Table 1), correlation analysis was carried out on questions Q6 and Q7 regarding the main reasons for parents to accept, reject or have doubts in order to examine the key variables, drivers and barriers to male HPV vaccination (Additional file 1. Correlation analysis of reasons to accept (Q6), have doubts or reject (Q7) HPV vaccination of sons).

**Table 1:** Parental acceptability of HPV vaccination of males, sons and daughters

Question	Results in %	UK	Germany	Italy	France
Q5. Would you want your son to be vaccinated against HPV?	*Yes*	75	72	70	49
		*p* = <0.001	*p* = <0.001	p = <0.001	
	*Yes, even if only partially reimbursed*	-	19	8	-
	*Yes, even if I pay myself*	7	2	2	-
		(450 £)	(450 €)	(515€)	
	*No*	3	20	15	34
			*p* = <0.01	*p* = <0.01	*p* = <0.01
	*Uncertain*	22	8	15	17
		*p* = <0.001		*p* = <0.001	*p* = <0.001
Q8. Do you think that boys should be vaccinated against HPV to reduce the overall transmission of HPV in the population?	*Yes*	85	73	70	82
		*p* = <0.1			*p* = <0.1
	*No*	2	20	19	14
			*p* = <0.001	*p* = <0.001	*p* = <0.001
	*Uncertain*	13	7	11	4
Q4. Attitudes towards the childhood immunisation programme	*Adherence*	89	77	77	78
		*p* = <0.001			
	*Partial adherence*	9	22	21	19
	*Non- adherence*	1	1	2	1
	*Uncertain*	1	-	-	-
Q3a. Will you let your daughter(s) aged below X (the eligible age) receive HPV vaccination when she/they reach(es) the eligible age?	*Yes*	92	67	66	65
		*p* = <0.001			
	*No*	2	13	11	14
	*Uncertain*	6	20	23	21
			*p* = <0.01	*p* = <0.01	*p* = <0.01
Q3b.Has/have your daughter(s) in the eligible age received HPV vaccination? Q3b2. If not, do you think she/they will be vaccinated?	*Yes*	67	36	75^a^	9
	*No*	27	64	25	91
	*Uncertain*	6	-	-	-
	*Intend to vaccinate*	67	36	-	56
Q3c. Has/have your daughter(s) older than X (the eligible age) received HPV vaccination?	*Yes*	44	44	51	47
	*No*	29	56	49	48
			*p* = <0.01	*p* = <0.01	*p* = <0.01
	*Uncertain*	27	-	-	5
Q3d. What is your attitude towards HPV vaccination of x year-old girls (asked only to parents with no daughters)	*For*	82 (vs.IT)	75	73	75
		*p* = <0.05			
	*Against*	2	17	14	11
	*Uncertain*	16	8	13	14
		*p* = <0.01		*p* = <0.05	*p* = <0.05

^a^ Low sample size: N = 4 respondents had 12 year-old daughters

- Implying that the response is not an option for the country in question or that no respondents answered this option

## Results

Most respondents (72–89 %) were mothers and the respondents were nationally representative with regard to varying educational and income levels, age, number and gender of children, urban/rural and region of residence (Table 1. Parental acceptability of HPV vaccination of males, sons and daughters).

Overall, parents were in favour of male HPV vaccination to reduce the transmission of HPV in society (70–85 %) and of their own sons (49–75 %). Number and gender of children in the household, gender of respondent, level of education and income were generally not significant variables related to acceptability. Exceptions were that, in Germany, region of residence was a significant variable, whereas town size was related to acceptability in Italy, and having a daughter played a minor role in France.

In all countries, the main correlates of favourable attitudes toward HPV vaccination of sons were positive attitudes toward other childhood vaccines in the NIP/NVC, and positive attitudes towards male HPV vaccination to reduce the overall transmission of HPV in society. Hence forward, we shall name parents in favour of HPV vaccination of their sons ‘*approvers’*, parents uncertain about HPV vaccination of their sons ‘*doubters’*, and parents against HPV vaccination of their sons ‘*rejecters’*. In all countries, doubters and approvers had similar participation rates in the NIP/NVC, whereas rejecters had significantly lower uptake of childhood vaccines.

### UK

Among the four countries, parents in the UK were the most favourable towards HPV vaccination of their sons (75 %) and toward generalised male HPV vaccination (85 %). Parents in the UK had the highest compliance with the NIP, the highest number of eligible daughters vaccinated against HPV, and of unvaccinated eligible daughters intended to be vaccinated in due time.

### Germany

In Germany, 72 % of parents were in favour of HPV vaccination of their sons. Acceptability was significantly higher in Northern (82 %) and Eastern Germany (91 %). Seventy percent were in favour of vaccinating males to reduce the transmission of HPV in society. Seventy-seven percent stated to have given their children all vaccines in the NVC. These figures were not matched by HPV vaccination rates among daughters, however. Only 36 % of eligible girls, and 44 % of older daughters, had been HPV vaccinated.

### Italy

In Italy, 70 % of parents were in favour of having their sons receive HPV vaccination. Parents living in mid-sized cities were significantly less favourable (55 %). 77 % of parents had fully complied with the NIP, and 73 % were in favour of general male HPV vaccination to reduce the transmission of HPV in the population. In Italy, 51 % of parents of daughters above the age of 12 stated that these had been vaccinated against HPV.

### France

In France, 49 % of the parents were in favour of HPV vaccination of theirs sons, while 34 % rejected. This acceptance rate is significantly lower than in the other three countries. In contrast, 78 % of French parents stated full compliance with the NVC, and 82 % were in favour of generalized male HPV vaccination. Only 9 % of parents stated that daughters in the eligible age bracket (11–14 year-old) received HPV vaccination which is unsurprising given the recently lowering of the targeted age group. 56 % intended eligible daughters to be vaccinated. The uptake among daughters aged >15 years was 47 %. Parents of daughters were more often doubters than parents without daughters.

### Pooled analysis

Given brief oral information on HPV-related disease in males, most parents in Germany, UK and Italy were in favour of HPV vaccination of their sons (70–75 %). In the UK, a school based vaccination programme plays a key role in the high uptake of all childhood vaccines in the NIP, and parental attitudes toward male HPV vaccination were correspondingly high. In Italy, attitudes toward HPV vaccination of sons were as favourable as attitudes towards female HPV vaccination and the NIP, in general. In Germany, parents mostly stated to be in favour of male HPV vaccination, but a high number of parents rejected HPV vaccination of their sons, particularly in Western Germany. HPV vaccination of daughters was low. In France, significantly less (49 %) parents were in favour of HPV vaccination of their sons despite favourable attitudes towards generalised male HPV vaccination. The low acceptance rates for HPV vaccination of sons matched the acceptance of daughters’ HPV vaccination (Table 2. Parents' reasons to accept, reject or have doubts about HPV vaccination of sons).

**Table 2:** Parents’ reasons to accept, reject or have doubts about HPV vaccination of sons

Unassisted question	UK	Germany	Italy	France
*Approvers (Q6)*	To protect my son against STD (59 %)	Both sexes should have equal rights to vaccination (52 %) *p* value <0.001	Both sexes should have equal rights to vaccination (65 %) *p* value <0.001	To protect my son from STD (63 %)
	To protect my son’s future partners (24 %)	I welcome any protection of my children against cancer (51 %)	Both sexes are equally responsible for preventing STD (53 %)	I welcome any protection of my children against cancer (25 %)
	I welcome all vaccines for children (23 %)	Both sexes are equally responsible for preventing STD (44 %)	My son is also at risk of HPV infection (50 %)	To protect my son’s future partners (19 %)
	My son is also at risk from HPV infection (19 %)	Fear of HPV related diseases if not vaccinated (42 %)	I welcome any protection against cancer (49 %)	If HPV vaccination is recommended by a HCP (13 %)
*Doubters (Q7)*	I don’t know enough about HPV vaccination (54 %)	I don’t know enough about HPV vaccination (69 %)	I don’t know enough about HPV related diseases (83 %)	I fear side effects (45 %)
	I don’t know enough about HPV related diseases (53 %)	I fear side effects (61 %)	I don’t know enough about HPV vaccination (67 %)	I don’t know enough about HPV related diseases (38 %)
	I fear side effects (25 %)	I don’t know enough about HPV related diseases (56 %)	I fear side effects (62 %)	Lack of recommendation from HCP (31 %)
	I prefer that my son makes his own decision later (13 %)	I am against (too many) vaccines (39 %)	Lack of recommendation from HPC (44 %)	I don’t know enough about HPV vaccination (28 %)
*Rejecters (Q7)*	It goes against my cultural/ religious beliefs (39 %)^a^	I fear side effects (64 %)	I fear side effects (76 %)	I fear side effects (46 %)
	Other (31 %)^b^	I don’t know enough about HPV vaccination (57 %)	I am against (too many) vaccines (73 %)	Lack of recommendation from HCP (23%)
	I fear side effects/my son is too young (23 % respectively)	I am against (too many) vaccines (54 %)	I don’t know enough about HPV related diseases (55 %)	I don’t know enough about HPV vaccination (21 %)
	I don’t know enough about HPV related diseases (15 %)	I don’t know enough about HPV related diseases (47 %)	I don’t know enough about HPV vaccination (51 %)	I am against (too many) vaccines (17 %)

^a^Note the low sample size for UK rejecters: 3 % of all UK respondents.

^b^Most ‘Other’ responses from the UK fit in preexisting response options and mainly dealt with vaccine safety and efficacy concerns, the vaccine being too ‘new’ to be considered safe, and the son being too young

In the UK and France, main reasons to be in favour of HPV vaccination of one’s own sons were to protect them from (any) STD (59/63 %, respectively). Next, some parents stated that they wanted to protect their sons’ future partners (24/19 %). In the UK, 23 % of parents said they welcomed all vaccines. In France, 25 % welcomed any protection against cancer.

A broader range of answers were given in Germany and Italy. Here, equal rights of HPV vaccination was the main reason (52 and 65 %, respectively), and shared responsibility to prevent STDs was also significantly more often stated as reasons to be favourable (44/53 %) than in the UK and France. In Germany and Italy, any protection against cancer was welcomed (by 51/49 %), and in Italy, 59 % said their son was also being at risk of HPV infection.

While fear of vaccination side effects and being against (too many) vaccines were main barriers to rejecters, and particularly widespread in Germany and France, lack of knowledge about HPV related diseases and HPV vaccination were significantly more frequent barriers to doubters in all countries. In France and Italy, lack of recommendation from HCPs was also important (please find in Additional file 1 the correlation coefficients of the correlation matrix for Q6 and Q7 regarding main reasons to accept, reject or have doubts about HPV vaccination of sons) (Table 3. Are there any people or authorities whose recommendations you particularly listen to in connection with health-related issues such as this vaccination).

**Table 3:** Are there any people or authorities whose recommendations you particularly listen to in connection with health-related issues such as this vaccination

Unassisted Question	UK	Germany	Italy	France
*Total (Q9)*	GP (46 %)	GP (72 %)	GP (75 %)	GP (69 %)
		*p* <0.001	*p* <0.001	*p* <0.001
	National Health Service (NHS) (30 %)	Pediatrician (45 %)	Pediatrician (66 %)	Family (12 %)
	Other (unspecified) (29 %)	Krankenkasse (sick fund) (24 %)	Azienda Sanitaria Locale (Local Health Unit, AZL) (19 %)	Uncertain (10 %)
	Uncertain (23 %)	Friends and family (23 % respectively )	Gynecologist (17 %)	Social media (8 %)
*Approvers (Q9)*	My GP (46 %)	GP (74 %)	GP (78 %)	GP (70 %)
		*p* <0.001	*p* <0.001	*p* <0.001
	NHS (31 %)	Pediatrician (48 %)	Pediatrician (65 %)	Family (13 %)
	Other (31 %)	Krankenkasse (sick fund) (30 %)	Azienda Sanitaria Locale (AZL) (23 %)	Uncertain (9 %)
	Uncertain (23 %)	Family members (25 %)	Gynecologist (20 %)	Social media (8 %)
*Doubters (Q9)*	GP (51 %)	GP (68 %)	Pediatrician (73 %)	GP (78 %)
		*p* <0.01		*p* <0.001
	NHS (30 %)	Pediatrician (45 %)	GP (70 %)	Pediatrician (9 %)
		*p* <0.001	*p* <0.001	
	Uncertain (22 %)	Friends (33 %)	National health authorities (29 %)	Family (8 %)
		*p* <0.05		
	Other (22 %)	Social media (28 %)	Azienda Sanitaria Locale (AZL) (14 %)	The public health authorities or a gynecologist (7 % respectively)
		*p* <0.05		
*Rejecters (Q9)*	Uncertain (46 %)	GP (66 %)	GP (67 %)	GP (62 %)
		*p* <0.001	*p* <0.001	*p* <0.001
	Other (39 %)	Pediatrician (36 %)	Pediatrician (61 %)	Uncertain (16 %)
	GP (15 %)	Family (23 %)	National health authorities (19 %)	Family (10 %)
	Social media (15 %)	Friends (11 %)	Gynecologist (16 %)	Social media (9 %)

In all countries, GPs were by far parents’ main source of advice regarding vaccination. In Germany and Italy, paediatricians also play a very important role, whereas in the UK, the NHS has great authority. In Germany and France, family, friends and media had some influence. In Italy, this applied to the Local Health Units (Azienda Sanitaria Locale). Doubters in all countries listened (at least) as much as approvers to GPs and paediatricians (and the NHS in the UK). In Germany, doubters also listened significantly more to friends and social media, however. Overall, rejecters listened less to HCPs and more to family and social media.

## Discussion

HPV infected men are at risk of developing HPV related diseases and they increase the risk of infection in women. A normalisation of HPV vaccination may improve uptake and herd-immunity in women as well as men [3, 16, 24, 33–36]. Besides the issue of HPV related morbidity and mortality in both sexes, ethical issues regarding social justice (considerations of MSM and disadvantaged populations) and gender equality should be considered [37, 38]. Today, a major challenge to HPV vaccination is to transform the image of a females-only cervical cancer vaccine to a universal childhood vaccine.

The present study had the strength of including a large number of decision-making parents of sons at a relevant age for HPV vaccination across four countries in the EU. The analysis allowed us to gain knowledge about drivers and barriers to parental attitudes towards male HPV vaccination and significant factors related to acceptability. As the study did not include clinical register data, however, intentions to let unvaccinated sons and daughters vaccinate against HPV may be over-stated; the same limitation applies to the stated compliance with the NIP/NVC including HPV vaccination of daughters. Furthermore, the data collection methods differed in the four countries lowering the comparative strength of the study. Answers from face-to-face interviews were more comprehensive, but the presence of an interviewer may have biased respondents towards more positive attitudes towards (HPV) vaccination. Responses given to the multiple-answer questions in the telephone interviews were fewer but likely to include the most pertinent issues to the respondents. As quota sampling is a non-random methodology implying that not every everyone gets a chance of participation, a selection bias may be involved. With face-to-face interviews, in particular, favourable respondents may me more likely volunteer to participate creating a possible bias towards positive attitudes. Finally, the sample sizes imply that some country specific sub-categories involve only few respondents thus lowering the strength of the cross-tab analysis.

Still, our results support previous findings that direct protection attributes is a central driver to parental acceptability of HPV vaccination of sons [11, 17, 33]. In addition, we found that many parents consider gender equality in health exceedingly important, and that given information about HPV vaccination and the total benefits of male HPV vaccination, parental acceptability rates are similar to vaccine acceptability for daughters [3, 38, 39].

Parents’ reasons to doubt or reject HPV vaccination of their sons differed significantly. Doubters particularly called for information about HPV related diseases and HPV vaccination. Rejecters had more pronounced concerns about side effects and vaccines, in general. A central means to augment the uptake of HPV vaccination may be to engage in efforts to improve awareness and knowledge about HPV, specifically, but also improving trust in vaccines, in general [40]. According to the ECDC (2012), parents and prescribers (HCP) need to be provided with appropriate evidence-based information on the benefits and risks of HPV immunisation.

Barriers to vaccination are not the same whether a person is hesitant, omitting or opposed for philosophical reasons [20]. Broadly speaking, barriers to a vaccine can be logistical and/or cognitive. Cognitive issues include knowledge and perceptions. Vaccine attitudes are related to perceptions of benefits and risks of the vaccine and the targeted diseases, and these are linked to local public health messages and media coverage [24]. Negative media coverage, not only of HPV vaccination, but also of vaccines against Hepatitis B in France, MMR in the UK, and the pandemic flu in 2009 contributed to create a fear of vaccination disproportional to the fear of the preventable diseases [20, 33, 41–44]. While anti-vaccination convictions are only found in 3–7 % of the population [45], hesitancy has become a major issue, as people increasingly seek answers to their concerns in order to balance the pros and cons of vaccination. This has created a growing need for objective information about vaccines from public health authorities and HCPs [33]. Local studies confirm our results that GPs and paediatricians play a major role in shaping parents’ perceptions of vaccines [16, 20, 21, 38, 43, 46–50].

While HPV vaccine knowledge is crucial, some scholars have argued that structural incentives and barriers (cost and accessibility) may have even greater impact on uptake [16]. The highest HPV vaccine uptake rates are found in countries with free school-based (opt-out) or national public health programmes such as the UK where 80 % of the target group have been vaccinated. In Italy, there is no school-based programme, yet some regions have obtained similar high coverage rates through extensive planning and action from Local Health Units, e.g., 80.7 % in the Basilicata region where parents of 12 year-old girls were called directly [51]. In France and Germany, it has been suggested that school-based programmes could also improve HPV vaccination uptake [49, 52]. In the UK, the NIP goes hand in hand with favourable attitudes toward vaccines, even when knowledge about them is poor, and the need for knowledge is low [24, 40]. Hence a possible explanation of why parents in the UK are not always aware of which and when specific vaccines are given. Much like in Denmark, the UK public is adapted to national vaccination policies. This implies that NIP vaccines are mostly trusted and considered important – while non-NIP vaccines are not. One study has shown that UK parents appreciate that the school-based programme enables them to abdicate responsibility to the authorities and that it creates equal health opportunities for children [45]. Outside schools, boys are generally less in contact with HCPs than girls [25].

## Conclusion

Two factors play a major role in HPV vaccination uptake: knowledge and structural incentives. When structural barriers are low, as in Italy and the UK, acceptability is high but knowledge does not necessarily follow. When structural barriers are higher, as in France and Germany, the need for knowledge and reassurance is higher. Independently of the chosen national vaccination strategy, however, parents and young people should be informed about HPV related diseases in males and the benefits and risks of accepting or rejecting HPV vaccination [53]. If HPV vaccination is to reach the optimal effectiveness in European populations, this poses a large responsibility on public health authorities and HCPs to inform the public. The education of HCPs about HPV and their direct recommendation to their patients is particularly crucial to parents in countries with opportunistic vaccination [54, 55].

## Additional file

Additional file 1:
**Correlation analysis of reasons to accept (Q6), have doubts or reject (Q7) HPV vaccination of sons.**

## Abbreviations

HPV: Human Papillomavirus
HCP: Health care professionals/providers
CC: Cervical cancer
ECDC: European Centre for Disease Control
UK: United Kingdom
NVC: National Vaccination Calendar
NIP: National Immunization Programme
CATI: Computer Assisted Telephone Interviews
CAPI: Computer Assisted Personal Interviews
INSEE: National Institute of Statistics and Economic Studies (France)
ONS: Office for National Statistics (UK)
ISTAT: Italian National Statistics Institute
STD: Sexually transmitted disease(s)
NHS: National Health Service (UK)
MMR: Measles, mumps and rubella (vaccine)
