# Supplementary material for: Parental attitudes towards male human papillomavirus vaccination: a pan-European cross-sectional survey
Source: BMC Public Health. 2015 Jul 8;15:624. doi: 10.1186/s12889-015-1863-6 (PMC4495645; doi:10.1186/s12889-015-1863-6)
Supplement: Additional file 1: — Correlation analysis of reasons to accept (Q6), have doubts or reject (Q7) HPV vaccination of sons. [file 12889_2015_1863_MOESM1_ESM.docx]

**Additional file 1. Correlation matrix of reasons to accept (Q6), have doubts or reject (Q7) HPV vaccination of sons**

***Q6*** *What are the main reasons for wanting your son(s) to receive the HPV vaccine?*

***Q7*** *What are the main reasons you do not want or are uncertain about your son(s) receiving the HPV vaccine?*
